# Genetic knockout and pharmacologic inhibition of neuronal nitric oxide synthase attenuate nerve injury-induced mechanical hypersensitivity in mice

**DOI:** 10.1186/1744-8069-3-29

**Published:** 2007-10-08

**Authors:** Yun Guan, Myron Yaster, Srinivasa N Raja, Yuan-Xiang Tao

**Affiliations:** 1 Department of Anesthesiology and Critical Care Medicine, Johns Hopkins University School of Medicine, 355 Ross, 720 Rutland Ave., Baltimore, Maryland 21205, USA

## Abstract

Neuronal nitric oxide synthase (nNOS) is a key enzyme for nitric oxide production in neuronal tissues and contributes to the spinal central sensitization in inflammatory pain. However, the role of nNOS in neuropathic pain remains unclear. The present study combined a genetic strategy with a pharmacologic approach to examine the effects of genetic knockout and pharmacologic inhibition of nNOS on neuropathic pain induced by unilateral fifth lumbar spinal nerve injury in mice. In contrast to wildtype mice, nNOS knockout mice failed to display nerve injury-induced mechanical hypersensitivity. Furthermore, either intraperitoneal (100 mg/kg) or intrathecal (30 μg/5 μl) administration of L-N^G^-nitro-arginine methyl ester, a nonspecific NOS inhibitor, significantly reversed nerve injury-induced mechanical hypersensitivity on day 7 post-nerve injury in wildtype mice. Intrathecal injection of 7-nitroindazole (8.15 μg/5 μl), a selective nNOS inhibitor, also dramatically attenuated nerve injury-induced mechanical hypersensitivity. Western blot analysis showed that the expression of nNOS protein was significantly increased in ipsilateral L_5 _dorsal root ganglion but not in ipsilateral L_5 _lumbar spinal cord on day 7 post-nerve injury. The expression of inducible NOS and endothelial NOS proteins was not markedly altered after nerve injury in either the dorsal root ganglion or spinal cord. Our findings suggest that nNOS, especially in the dorsal root ganglion, may participate in the development and/or maintenance of mechanical hypersensitivity after nerve injury.

## Background

Considerable evidence has shown that nitric oxide (NO) acts as an important mediator in the peripheral and central nervous systems and functions in a wide variety of physiologic and pathophysiologic processes, such as neurotransmission, synaptic plasticity, neuroprotection, neurotoxicity, and pathologic pain [1-3]. NO is synthesized by three well-characterized isoforms of NO synthase (NOS): neuronal NOS (nNOS), endothelial NOS (eNOS), and inducible NOS (iNOS). Under pathologic conditions, these three NOS isoforms could be upregulated in nervous tissues [4-6]. Thus, the pathophysiologic functions of NO in the nervous system may be regulated by the expression and activity of one, two, or all three NOS isoforms.

Neuronal NOS is expressed in the neurons and produces predominantly NO in neuronal tissues [1]. The contribution of nNOS-synthesized NO to nociceptive processing has been characterized in several inflammatory pain models [2]. Peripheral inflammation induced by formalin or complete Freund's adjuvant increases nNOS (but not eNOS or iNOS) expression in the spinal cord [5,7,8] and dorsal root ganglion (DRG) [9]. Systemic or intrathecal administration of nonspecific NOS inhibitors or selective nNOS inhibitors reduces the exaggerated pain in animals after formalin-, carrageenan-, or complete Freund's adjuvant-induced peripheral inflammation [5,9-15]. Moreover, targeted disruption of the nNOS gene attenuates carrageenan- and complete Freund's adjuvant-induced thermal and mechanical pain hypersensitivity in mice [5,15], although nNOS knockout mice were reported to display intact formalin-induced nociceptive behaviors [16]. These observations indicate that nNOS at the spinal cord level may play a critical role in the central mechanism of inflammatory pain.

In addition to inflammation, peripheral nerve injury also causes clinically relevant persistent or chronic pain. Although nerve injury-induced neuropathic pain has some unique characteristics in regard to pathogenesis, central mechanisms, and treatment compared to inflammatory pain [17], these two types of persistent pain may share some intracellular signaling pathways in their central mechanisms [18]. However, the exact role of nNOS in neuropathic pain, especially at the spinal cord level, is unclear. Peripheral nerve injury increased expression of both mRNA and protein for nNOS in the DRG, but not in spinal cord [19-22]. Although systemic or spinal treatment with specific and nonspecific nNOS inhibitors has been shown to block neuropathic pain [23-27], two investigators reported that systemic or intrathecal administration of specific and nonspecific nNOS inhibitors had no effect on nerve injury-induced allodynia [19,28]. It is evident that these pharmacologic results are conflicting. In the current study, by combining a genetic strategy (using nNOS knockout mice) with a pharmacologic approach (using selective and nonspecific nNOS inhibitors), we determined the functional role of nNOS in chronic neuropathic pain induced by L_5 _spinal nerve injury in mice. In addition, we examined the expression of nNOS, as well as iNOS and eNOS, in DRG and spinal cord after spinal nerve injury.

## Results

### Effect of nNOS knockout on spinal nerve injury-induced mechanical hypersensitivity

**Figure 1:**
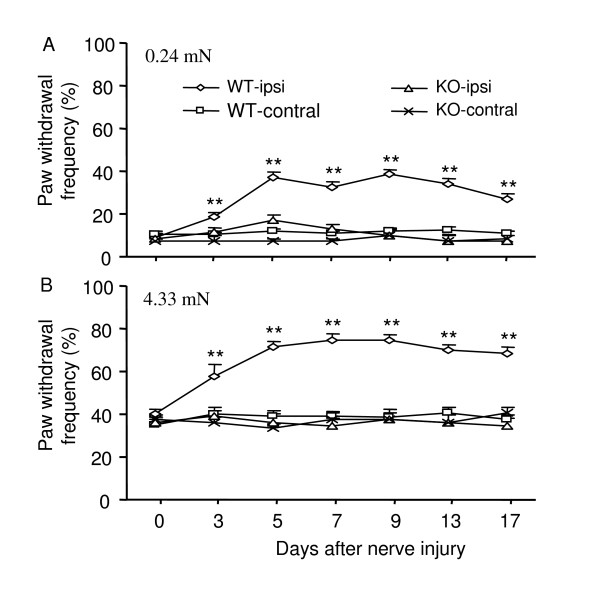
Effect of targeted disruption of the nNOS gene on nerve injury-induced neuropathic pain. L_5 _spinal nerve injury produced a significant increase in paw withdrawal frequencies in response to 0.24 mN (low intensity) (A) and 4.33 mN (moderate intensity) (B) mechanical stimuli on the ipsilateral (ipsi) side in wildtype (WT) mice. The nNOS knockout (KO) mice displayed impaired mechanical pain hypersensitivity on the ipsilateral side (A, B). No significant changes in paw withdrawal frequencies were seen on the contralateral (contral) side after L_5 _spinal nerve injury in either WT or nNOS KO mice. **P < 0.01 *vs *corresponding time points in the WT mice.

Consistent with our previous studies [29,30], the fifth lumbar spinal nerve injury produced long-term mechanical hypersensitivity on the ipsilateral hind paw in wildtype (WT) mice (*n *= 12). The application of 0.24 mN (low intensity) and 4.33 mN (moderate intensity) von Frey filaments to the plantar side of the hind paw ipsilateral to the nerve injury elicited significant increases in paw withdrawal frequencies, as compared to pre-injury baseline values, a behavioral indication of mechanical hypersensitivity. This mechanical hypersensitivity appeared on day 3, reached a peak level between days 5 and 7, and persisted for at least 17 days post-nerve injury (Fig. 1A, B). The baseline withdrawal responses to mechanical stimuli were similar in WT and nNOS knockout mice (Fig. 1A, B), but nNOS knockout mice (*n *= 10) did not display mechanical hypersensitivity following spinal nerve injury. Compared to the baseline level, no significant increases in withdrawal frequencies were observed in response to mechanical stimuli in the knockout mice (*P *> 0.05; Fig. 1A, B). As expected, no significant changes in paw withdrawal responses to mechanical stimuli were seen on the contralateral hind paw in either WT or nNOS knockout mice following spinal nerve injury (Fig. 1A, B).

### Effect of systemic administration of a nonspecific NOS inhibitor on nerve injury-induced mechanical hypersensitivity

**Figure 2:**
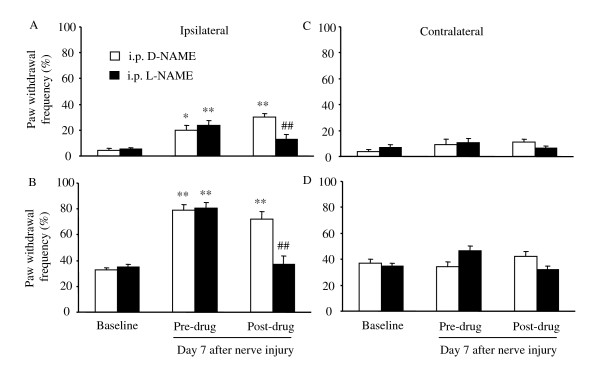
Effect of intraperitoneal (i.p.) injection of L-NAME or D-NAME on mechanical pain hypersensitivity on day 7 after L_5 _spinal nerve injury in WT mice. Paw withdrawal responses to 0.24 mN (A and C) and 4.33 mN (B and D) mechanical stimuli on the ipsilateral (A and B) and contralateral (C and D) sides. *P < 0.05, **P < 0.01 *vs *the corresponding baseline. ##*P *< 0.01 *vs *the post-drug, D-NAME-treated group.

We examined the effect of systemic inhibition of NOS on nerve injury-induced mechanical pain hypersensitivity in WT mice. A nonspecific NOS inhibitor, L-N^G^-nitro-arginine methyl ester (L-NAME) and its inactive enantiomer, D-NAME, were administered intraperitoneally on day 7 post-nerve injury. Intraperitoneal injection of D-NAME failed to produce a significant effect on nerve injury-induced increases in ipsilateral paw withdrawal frequencies in response to low- and moderate-intensity mechanical stimuli (*n *= 9; Fig. 2A, B). However, intraperitoneal injection of L-NAME significantly reversed nerve injury-evoked mechanical hypersensitivity on the ipsilateral side (*n *= 11; Fig. 2A, B). The average paw withdrawal frequencies to low- and moderate-intensity mechanical stimuli after administration of L-NAME were reduced by 58% and 48%, respectively, compared to the values in the D-NAME-treated groups (*P *< 0.01). As expected, neither L-NAME nor D-NAME at the doses used affected contralateral paw withdrawal responses to mechanical stimuli (Fig. 2C, D).

### Effect of intrathecal injection of nonspecific and specific nNOS inhibitors on nerve injury-induced mechanical hypersensitivity

**Figure 3:**
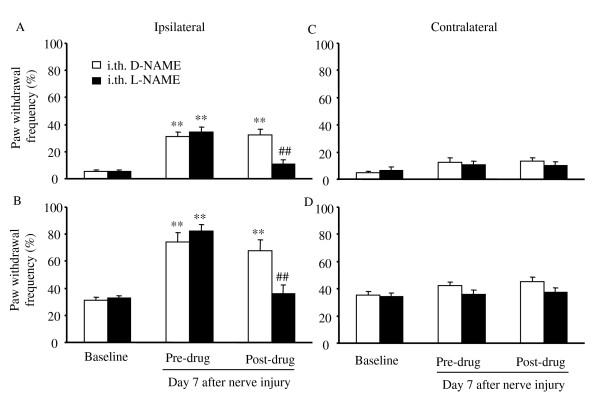
Effect of intrathecal (i.th.) injection of L-NAME or D-NAME on mechanical pain hypersensitivity on day 7 after L_5 _spinal nerve injury in WT mice. Paw withdrawal responses to 0.24 mN (A and C) and 4.33 mN (B and D) mechanical stimuli on the ipsilateral (A and B) and contralateral (C and D) sides. **P < 0.01 *vs *the corresponding baseline. ##*P *< 0.01 *vs *the post-drug, D-NAME-treated group.

**Figure 4:**
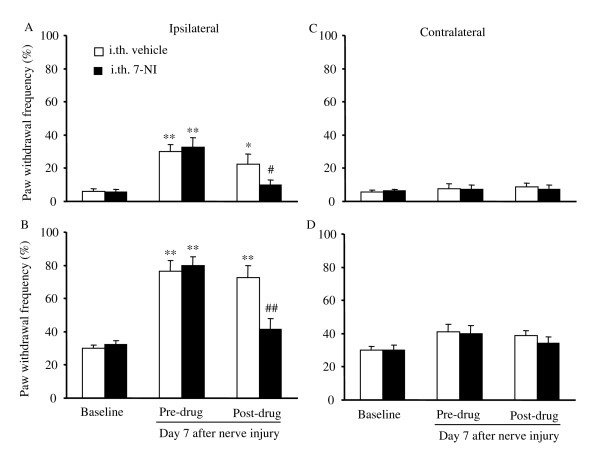
Effect of intrathecal injection of 7-NI on mechanical pain hypersensitivity on day 7 after L_5 _spinal nerve injury in WT mice. Paw withdrawal responses to 0.24 mN (A and C) and 4.33 mN (B and D) mechanical stimuli on the ipsilateral (A and B) and contralateral (C and D) sides. *P < 0.05, **P < 0.01 *vs *the corresponding baseline. ##*P *< 0.01 *vs *the post-drug, vehicle-treated group.

To define the role of NOS, especially nNOS, in neuropathic pain at the spinal cord level, we next examined the effect of intrathecally injected L-NAME or 7-nitroindazole (7-NI), a specific nNOS inhibitor, on nerve injury-induced mechanical hypersensitivity 7 days after spinal nerve injury in WT mice. In D-NAME-treated (*n *= 10) or vehicle (20% DMSO)-treated (*n *= 8) groups, withdrawal frequencies to low- and moderate-intensity mechanical stimuli applied to the ipsilateral hindpaw were significantly higher than those at baseline (*P *< 0.01; Figs. 3A, B, and 4A, B). In contrast, intrathecal injection of L-NAME significantly reversed nerve injury-induced mechanical hypersensitivity (*n *= 12; Fig. 3A, B). Compared to those in D-NAME-treated animals, paw withdrawal frequencies in response to low- and moderate-intensity mechanical stimuli were reduced by 66% and 47%, respectively (*P *< 0.01). Importantly, intrathecal 7-NI (*n *= 7) produced inhibitory effects similar to L-NAME on the nerve injury-induced mechanical pain hypersensitivity (Fig. 4A, B). Paw withdrawal frequencies were reduced by 56% and 43% of the values in the vehicle-treated groups in response to low- and moderate-intensity mechanical stimuli, respectively (*P *< 0.05). Neither 7-NI nor L-NAME altered the responses to mechanical stimuli on the contralateral hindpaw (Figs. 3C, D, and 4C, D).

### Effect of spinal nerve injury on nNOS expression in the DRG and spinal cord

**Figure 5:**
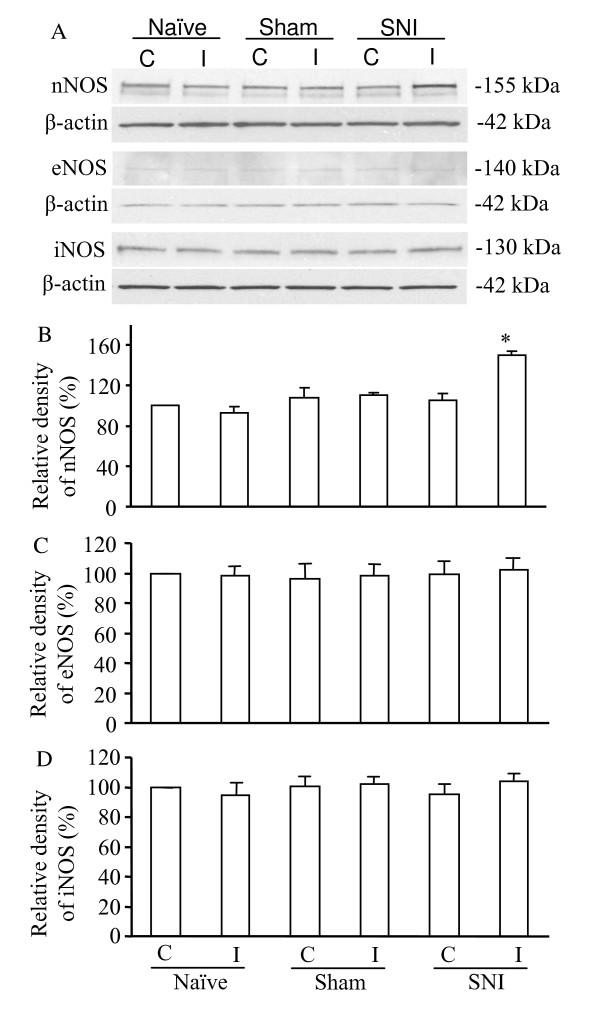
Expression of nNOS, eNOS, and iNOS in the ipsilateral (I) and contralateral (C) L_5 _dorsal root ganglia in naïve WT mice and WT mice 7 days after sham surgery or L_5 _spinal nerve injury. (A) Representative examples of Western blots. β-actin was used as a loading control. (B-D) Statistical summary of the densitometric analysis expressed relative to the contralateral side in naïve mice. *P < 0.05 *vs *the corresponding side in naïve mice or the sham-operated group.

**Figure 6:**
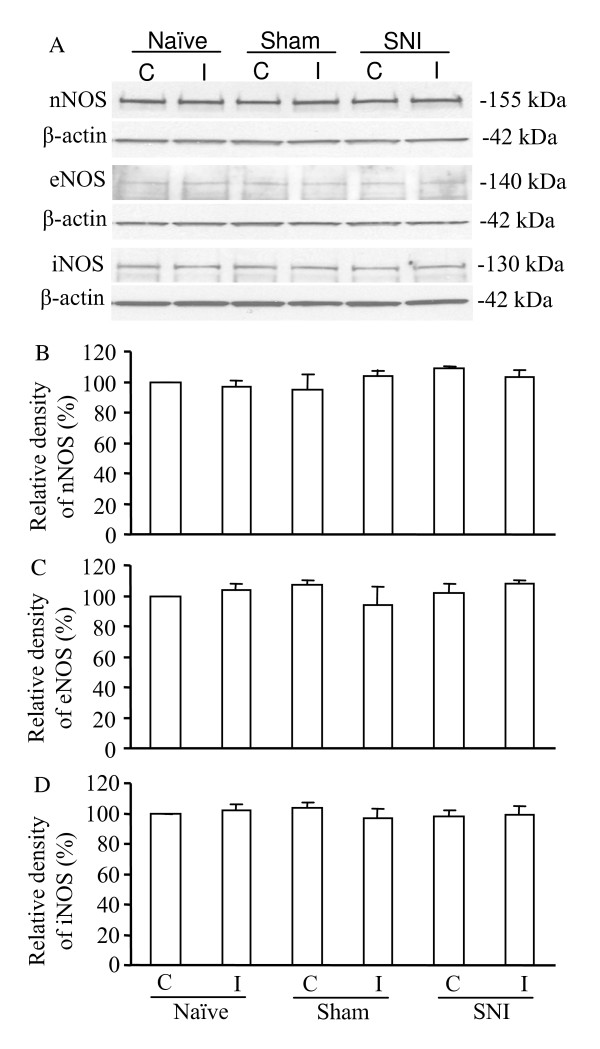
Expression of nNOS, eNOS, and iNOS in the ipsilateral (I) and contralateral (C) dorsal horn of L_5 _spinal cord in naïve WT mice and WT mice 7 days after sham surgery or L_5 _spinal nerve injury. (A) Representative examples of Western blots. β-actin was used as a loading control. (B-D) Statistical summary of the densitometric analysis expressed relative to the contralateral side in naïve mice.

Finally, we examined whether peripheral spinal nerve injury altered the expression of nNOS, iNOS, or eNOS in the DRG and spinal cord of WT mice. We found that nNOS in the ipsilateral (but not in the contralateral) fifth DRG was increased after the L_5 _spinal nerve injury (Fig. 5). Quantification showed that nNOS protein levels in the ipsilateral DRG tissues on day 7 post-nerve injury (*n *= 16 mice) were 1.6-fold greater than those from naïve mice (*n *= 16 mice; *P *< 0.05) and 1.4-fold more than those on day 7 post-sham surgery (*n *= 16 mice; *P *< 0.05). Interestingly, the amount of nNOS protein in the ipsilateral and contralateral L_5 _dorsal horn on day 7 post-nerve injury (*n *= 16 mice) was similar to that from naïve mice (*n *= 16 mice) and mice 7 days after sham surgery (*n *= 16 mice; *P *> 0.05; Fig. 6). In addition, L_5 _spinal nerve injury did not change the expression level of nNOS in either ipsilateral or contralateral L_4 _DRG and L_4 _dorsal horn at day 7 post-nerve injury (data not shown). The expression levels of eNOS and iNOS in the DRG and spinal cord were not markedly different on day 7 post-nerve injury than those of the sham groups or naïve mice (*n *= 16 mice/group;*P *> 0.05; Figs. 5, 6).

## Discussion

The present study provides the first evidence that targeted disruption of the nNOS gene significantly attenuates nerve injury-induced mechanical hypersensitivity. Moreover, nerve injury-induced mechanical hypersensitivity in WT mice was significantly inhibited by systemic and intrathecal administration of L-NAME and by intrathecal treatment with 7-NI. Peripheral nerve injury increased the expression of nNOS in the DRG, although it did not alter the expression of nNOS in the spinal cord. Our findings suggest that nNOS, especially in the DRG, may play a critical role in nerve injury-induced mechanical hypersensitivity.

Conflicting pharmacological evidence has been reported regarding the effects of systemic or spinal treatment with specific or non-specific NOS inhibitors on neuropathic pain. Meller et al. [24] first reported that intrathecal administration of L-NAME (20 nmol) on day 3 after loose ligation of the sciatic nerve blocked the thermal hyperalgesia in rats for a period of 2 h. A subsequent study also demonstrated that injecting 3-bromo-7-NI (a specific nNOS inhibitor, 10 mg/kg) intramuscularly into the mid-thigh region daily for 7 days beginning on day 7 post-surgery significantly alleviated nerve injury-induced thermal hyperalgesia from weeks 5 and 6 onward in rats [25]. Yoon et al. [26] further showed that both mechanical and cold allodynia induced by tight ligature of the left L_5 _and L_6 _spinal nerves were dose dependently suppressed during the maintenance phases (1 to 3 weeks post-ligature) by intraperitoneal L-NAME (10, 50, 100, and 200 μM/kg). In support of these findings, our pharmacological study showed that spinal nerve injury-induced mechanical hypersensitivity in mice could be dramatically attenuated by systemically (100 mg/kg) and intrathecally (30 μg) injected L-NAME and by intrathecally injected 7-NI (8.15 μg) on day 7 post-nerve injury (Figs. 2, 3, 4). Interestingly, Yamamoto and Shimoyama [27] found that the development of thermal hyperesthesia evoked by sciatic nerve constriction injury was significantly delayed by intrathecal pre-treatment (10 min before injury), but not post-treatment (15 min after injury), with NOS inhibitors N^ω^-nitro-L-arginine (30 μg) and L-NAME (100 μg). However, Pan et al. [28] reported that allodynia induced by tight ligation of the left L_5 _and L_6 _spinal nerves was not affected by intrathecal injection of either N^G^-monomethyl-arginine (a non-specific NOS inhibitor, 30 μg) or 1-(2-trifluoromethylphenyl) imidazole (a nNOS inhibitor, 30 μg) at the third or fourth week post-nerve injury. Additionally, Luo et al. [19] observed that neither pre-surgical (once daily for 6 days beginning 30 min before surgery) nor post-surgical (days 11 through 13) 7-NI (50 mg/kg) given subcutaneously or intraperitoneally affected the development or maintenance of tactile allodynia evoked by tight ligature of the left L_5 _and L_6 _spinal nerves in rats. The reason for the discrepancies among these pharmacological studies is unclear but may be related to the drug potency, delivery method, dose, and administration times. It is worth noting that only one dose of a nonspecific or specific nNOS inhibitor was administered by Luo et al. [19] and Pan et al [28]. It is possible that the higher doses used in these two studies might have had a significant inhibitory effect on nerve injury-induced neuropathic pain. Our nNOS knockout mouse study clearly demonstrated that deficiency of nNOS significantly blunted spinal nerve injury-induced mechanical hypersensitivity (Fig. 1). In addition, nNOS knockout mice exhibited impaired nerve injury-induced thermal hypersensitivity (data not shown). Although it has been reported that spinal eNOS is upregulated in nNOS KO mice [5], it appears that this upregulation does not fully compensate for nNOS function in neuropathic pain. Thus, our genetic knockout and pharmacologic studies support the notion that nNOS, particularly at the spinal cord level, may contribute to the mechanisms that underlie the development and/or maintenance of neuropathic pain.

Peripheral nerve injury insult has distinct effects on nNOS expression in the DRG and dorsal horn. Under normal conditions, the DRG contains only a few neurons that exhibit nNOS labeling, the majority of which are very small, whereas spinal dorsal horn exhibits intense nNOS labeling in several neuronal types, especially in the superficial dorsal horn [20]. It has been reported that constriction of the common sciatic nerve in rats increased nNOS expression in the ipsilateral L_4_-L_6 _DRG, but not in spinal segments L_4_-L_6 _[20]. The increased level of nNOS protein in the DRG was seen in the small, medium, and large cell bodies [20]. Likewise, tight, unilateral ligature of the rat L_5 _and L_6 _spinal nerves increased nNOS mRNA in ipsilateral L_5/6 _DRG neurons, but not in dorsal horn, beginning 1 day after nerve ligation and lasting for at least 13 weeks [19]. A corresponding increase in DRG (but not spinal cord) nNOS protein was also observed and localized mainly to small and occasionally medium-sized sensory neurons [19,21]. Consistent with these findings in rats, our study showed that unilateral injury of the fifth spinal nerve in mice also increased expression of nNOS protein in the ipsilateral fifth DRG, but not in dorsal horn. An increase in the DRG NOS catalytic activity and a decrease in spinal NOS catalytic activity have been reported following peripheral nerve injury [20,22]. The evidence described above indicates that peripheral nerve injury up-regulates expression and activity of nNOS in small DRG neurons and down-regulates its expression and activity in the spinal cord. Such adaptations suggest that the increased nNOS in the DRG may be involved in the mechanism underlying the development and/or maintenance of neuropathic pain. It has been demonstrated that intrathecally administered agents can directly affect activities of DRG proteins in pain processing in addition to affecting activities of these proteins in spinal cord neurons [31]. It is very likely that the pharmacologic effects of specific and non-specific NOS inhibitors in the present study were mediated through their actions on the DRG nNOS, although our data could not exclude their effects on spinal cord nNOS.

Consistent with previous reports [5,31,32], our study detected basal expression of eNOS and iNOS proteins in the spinal cord and DRG of mice under normal conditions. However, the expression of neither was altered in the spinal cord or DRG on day 7 post-nerve injury. It should be noted that we did not examine iNOS expression at the ligature site or at other time points post-nerve injury. Previous studies showed that local expression of iNOS was induced at 3 days and persisted for at least 26 days at the constriction and distal sites following chronic constriction partial nerve injury [33-36]. Systemic and topical administration of specific or non-specific iNOS inhibitors reversed nerve hyperaemia proximal and distal to the constriction [35]. Targeted disruption of the iNOS gene slowed Wallerian degeneration of myelinated fibers and delayed regeneration of myelinated and smaller caliber fibers in a chronic constriction partial nerve injury model [35]. Slowed nerve degeneration is associated with normal initiation but delayed expression of neuropathic pain [35], suggesting that local induction of iNOS expression may be important in the pathogenesis of nerve injury-induced neuropathic pain. Thus, our data do not exclude the possibility that iNOS at the ligature site might play a role in the development of neuropathic pain.

The mechanism by which DRG NOS-synthesized NO contributes to the development and/or maintenance of neuropathic pain is unclear. A gaseous molecular, NO diffuses out from the DRG neurons or primary afferent terminals and stimulates guanylyl cyclase in neighboring DRG or dorsal horn neurons to form cyclic guanosine monophosphate (cGMP) [2]. cGMP activates several intracellular processes, including cGMP-dependent protein kinase (PKG), ion channels, and phosphodiesterases (PDEs) [37]. PKG is present in the small DRG neurons and the superficial dorsal horn [38,39]. Sung et al. [40] showed that PKG was activated in peripheral terminals of the DRG neurons and retrogradely transported to the DRG after peripheral nerve injury and inflammation; however, intrathecal injection of a PKG inhibitor did not affect nerve injury-induced mechanical hypersensitivity [41]. Interestingly, intraplantar injection of a PDE-5 inhibitor significantly reduced mechanical hypersensitivity in diabetic neuropathy. This effect could be reversed by inhibition of NOS and guanylyl cyclase [42]. In addition, NO modulates the DRG Ca^2+ ^and Na^+ ^channels via cGMP-dependent pathways [43-45]. Therefore, it is very likely that DRG NOS-synthesized NO contributes to the mechanism that underlies the development and/or maintenance of neuropathic pain by activating PDEs and/or ion channels, but not by activating PKG, in the DRG and spinal cord.

In summary, we have provided genetic evidence that the deficiency of nNOS protein impairs mechanical hypersensitivity during the development and maintenance of neuropathic pain. Pharmacologic studies further demonstrated that systemic or spinal administration of specific and non-specific nNOS inhibitors attenuates nerve injury-induced mechanical hypersensitivity. Moreover, peripheral nerve injury up-regulates nNOS expression in the DRG but not in the spinal cord. These findings suggest that the DRG nNOS may play a role in the mechanism underlying neuropathic pain.

## Methods

### Animal preparation

The nNOS knockout mice (C57BL/6J background) were purchased from Jackson Laboratories (Bar Harbor, ME, USA), cross-bred with C57BL/6J WT mice in our laboratory, and kept on a standard 12-h light/dark cycle, with water and food pellets available ad libitum. The genomic status of each mouse was checked with the use of reverse transcriptase-PCR and Western blotting [5]. Male and female nNOS knockout mice are viable and fertile with normal appearance, as described before [5,46]. Male mice weighing 25–30 g were used, and the WT littermates were used as controls. To minimize intra- and inter-individual variability of behavioral outcome measures, animals were trained for 1–2 days before behavioral testing was performed. Animal experiments were conducted with the approval of the Animal Care and Use Committee at Johns Hopkins University and were consistent with the ethical guidelines of the National Institutes of Health and the International Association for the Study of Pain. All efforts were made to minimize animal suffering and to reduce the number of animals used.

### Experimental drugs

L-NAME and D-N^G^-nitro-arginine methyl ester (D-NAME) were purchased from Alexis Biochemicals (San Diego, CA, USA). 7-NI was purchased from Calbiochem Biosciences (La Jolla, CA, USA). 7-NI was dissolved in 20% dimethylsulfoxide (DMSO); all other drugs were dissolved in saline.

### Intrathecal injection

Intrathecal injection was performed under brief isoflurane (1.5%) anesthesia to reduce stress, as described previously [5]. Briefly, a 30-gauge, 0.5-inch needle connected to a 10-μl syringe was inserted into one side of the L_5 _or L_6 _spinous process at an angle of approximately 20° above the vertebral column and slipped into the groove between the spinous and transverse processes. While the angle of the syringe was decreased to approximately 10°, the needle was moved carefully forward to the intervertebral space. A tail flick indicated that the tip of the needle was inserted into the subarachnoid space. Total volume of the injected solution was 5 μl. Mice were completely recovered (that is, were spontaneously active) within 3–5 min after injection.

### Spinal nerve injury-induced neuropathic pain model in mice

The spinal nerve injury model was produced in mice as described previously [30]. In brief, the mice were anesthetized with isoflurane and placed in a prone position. The left paraspinal muscles were separated from the spinous processes at the L_4_-S_2 _levels under aseptic conditions, and the left L_5 _spinal nerve was isolated, tightly ligated with a silk thread (7-0), and transected just distal to the ligature. In a control sham group, the surgical procedure was identical to that described above, except that the left L_5 _spinal nerve was not ligated and transected. The mice were returned to their cages and observed for any signs of motor deficits. None of the mice showed motor dysfunction after surgery. Behavioral tests described below were performed 1 day before spinal nerve injury and 3, 5, 7, 9, 13, and 17 days after spinal nerve injury.

To examine the effects of systemic administration of NOS inhibitors on the maintenance of L_5 _spinal nerve injury-induced mechanical hypersensitivity, WT mice received intraperitoneal injection of L-NAME (100 mg/kg), D-NAME (100 mg/kg), or saline on day 7 after spinal nerve injury. To further define the role of NOS at the spinal cord level in spinal nerve injury-induced mechanical hypersensitivity, the WT mice received an intrathecal injection of L-NAME (30 μg/5 μl), D-NAME (30 μg/5 μl), 7-NI (8.15 μg/5 μl), or vehicle (20% DMSO or saline, 5 μl) on day 7 after spinal nerve injury. Behavioral tests were performed as described below 1 day before spinal nerve injury, 1 h before drug administration, and 20 min after drug administration on day 7 post-nerve injury. The doses and duration of drug administration were based on our previous studies and those of others [5,14,47,48].

### Behavioral testing

To measure paw withdrawal responses to repeated mechanical stimuli, each mouse was placed in a Plexiglas chamber on an elevated mesh screen. Two calibrated von Frey monofilaments (0.24 and 4.33 mN; Stoelting Co., Wood Dale, IL, USA) were used. Each von Frey filament was applied to the hind paw for approximately 1 sec, and each stimulation was repeated 10 times to both hind paws. The occurrence of paw withdrawal in each of these 10 trials was expressed as a percent response frequency [(number of paw withdrawals/10 trials) × 100 = % response frequency], and this percentage was used as an indication of the amount of paw withdrawal. Experimenters were blinded to mouse genotype and drug assignment.

### Western blot analysis

WT mice were sacrificed by decapitation on day 7 after spinal nerve injury or sham surgery. Naïve mice were used as controls. The fourth and fifth lumbar ipsilateral and contralateral DRGs and spinal dorsal horns were removed. All tissues were quickly frozen in liquid nitrogen and stored at -80°C for later use. Because of the small size of the unilateral fifth DRG and dorsal horn, the DRGs from four mice and dorsal horns from two mice were pooled together to obtain enough protein for Western blot analysis. Frozen tissues were homogenized in buffer (10 mM Tris-HCl, 5 mM MgCl_2_, 2 mM EGTA, 1 mM phenylmethylsulfonyl fluoride, 1 μM leupeptin, 2 μM pepstatin A, 1 mM dithiothreitol). The crude homogenate was centrifuged at 4°C for 15 min at 700 × g. The supernatants (100 μg) were heated for 5 min at 98°C and then loaded onto 4% stacking/7.5% separating SDS-polyacrylamide gels. The proteins were electrophoretically transferred onto nitrocellulose membrane, blocked with 3% nonfat dry milk, and subsequently incubated overnight at 4°C with polyclonal rabbit anti-nNOS antibody (1:1,000, BD Transduction Laboratories, San Diego, CA, USA), polyclonal rabbit anti-eNOS antibody (1:1,000, BD Transduction), polyclonal rabbit anti-iNOS antibody (1:1,000, BD Transduction), or monoclonal mouse anti-β-actin antibody (1:3,000, Sigma, St. Louis, MO, USA). β-actin was used as a loading control. The proteins were detected with horseradish peroxidase-conjugated anti-rabbit or anti-mouse secondary antibodies and visualized by chemiluminescence reagents provided with the ECL kit (Amersham Pharmacia Biotech, Piscataway, NJ, USA) and exposure to film. The intensity of blots was quantified with densitometry. The blot density from the contralateral DRG or dorsal horn in the naïve mice was set as 100%. The relative density values of the ipsilateral DRG or dorsal horn in the naïve mice and from ipsilateral or contralateral DRG or dorsal horn in the spinal nerve-injured or sham-operated group were determined by dividing the optical density values from these groups by a value from the contralateral DRG or dorsal horn in the naïve mice.

### Statistical analysis

Data from the behavioral tests and Western blots were expressed as mean ± SEM and analyzed with a one-way or two-way analysis of variance (ANOVA). When ANOVA showed significant differences, pairwise comparisons between means were tested by the post hoc Tukey method. A pairwise *t*-test was used to determine the significant differences of means for comparisons between two groups. Significance was set at *P *< 0.05. All statistical analyses were performed using the SigmaStat statistical software.
